# Participation of lipids in the tumor response to photodynamic therapy and its exploitation for therapeutic gain

**DOI:** 10.1016/j.jlr.2024.100729

**Published:** 2024-12-14

**Authors:** Mladen Korbelik, Michal Heger, Albert W. Girotti

**Affiliations:** 1 Department of Integrative Oncology, BC Cancer, Vancouver, BC, Canada; 2 Jiaxing Key Laboratory for Photonanomedicine and Experimental Therapeutics, Department of Pharmaceutics, College of Medicine, Jiaxing University, Jiaxing, Zhejiang, P. R. China; 3 Department of Pharmaceutics, Utrecht Institute for Pharmaceutical Sciences, Utrecht University, Utrecht, the Netherlands; 4 Membrane Biochemistry and Biophysics, Department of Chemistry, Faculty of Science, Utrecht University, Utrecht, the Netherlands; 5 Department of Biochemistry, Medical College of Wisconsin, Milwaukee, WI, USA

**Keywords:** lipid peroxidation, tumor lipid metabolism, antitumor lipids, oxidative damage, oxidative stress, repair mechanisms, membrane dysfunction, lipid signaling, cancer treatment

## Abstract

Hydroperoxides of unsaturated membrane lipids (LOOHs) are the most abundant non-radical intermediates generated by photodynamic therapy (PDT) of soft tissues such as tumors and have far longer average lifetimes than singlet oxygen or oxygen radicals formed during initial photodynamic action. LOOH-initiated post-irradiation damage to remaining membrane lipids (chain peroxidation) or to membrane-associated proteins remains largely unrecognized. Such after-light processes could occur during clinical oncological PDT, but this is not well-perceived by practitioners of this therapy. In general, the pivotal influence of lipids in tumor responses to PDT needs to be better appreciated. Of related importance is the fact that most malignant tumors have dramatically different lipid metabolism compared with healthy tissues, and this too is often ignored. The response of tumors to PDT appears especially vulnerable to manipulations within the tumor lipid microenvironment. This can be exploited for therapeutic gain with PDT, as exemplified here by the combined treatment with the antitumor lipid edelfosine.

Photodynamic therapy (PDT) is a light energy-utilizing clinically established modality for the eradication of cancers and nonmalignant lesions and is being explored for numerous other indications (Fig. 1A–F) (7). The light absorption is mediated by photosensitizing drugs (photosensitizers, PSs; Fig. 1G) administered to patients before the onset of illumination. The ablation of targeted tumors is instigated by a photochemical reaction prevalently initiated by photo-excited PS molecules interacting with molecular oxygen to generate cytotoxic oxidants, including singlet oxygen and other reactive oxygen species (ROS) (8). In addition to direct lethal effect by necrosis, apoptosis, or other programmed cell death pathways, PDT-treated tumors are destroyed indirectly as a consequence of vascular damage (hypoxia and metabolic catastrophe) or elicited antitumor immune responses (immunological cell death) (7, 8).

**Fig. 1:**
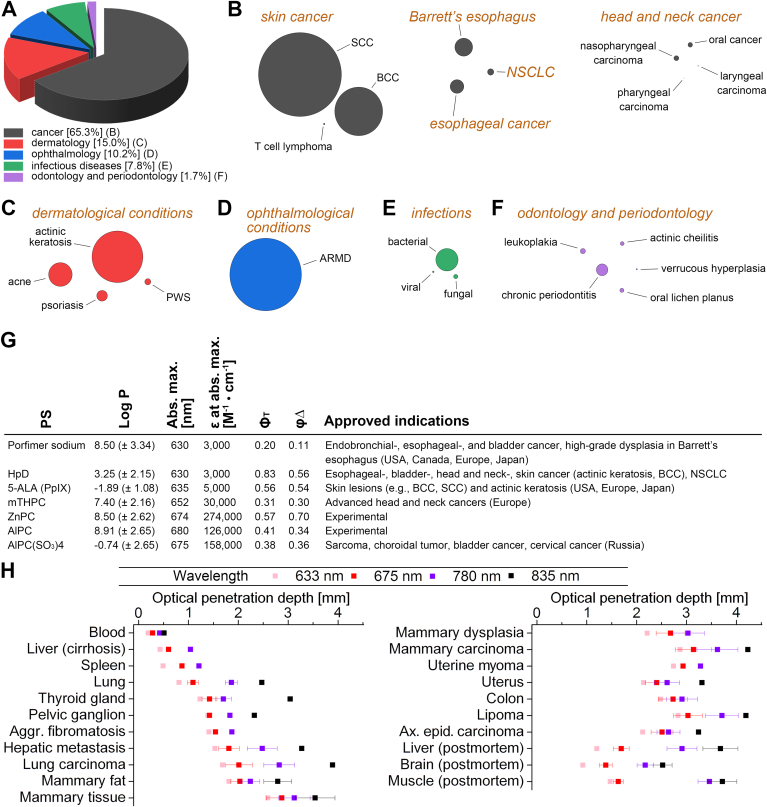
A: Global overview of published peer-reviewed research papers on photodynamic therapy (PDT), stratified into the 5 largest research fields, with substratifications for the field of cancer (B), dermatology (C), ophthalmology (D), infectious diseases (E), and odontology and periodontology (F). The circle diagrams are sized in proportion to the number of retrieved publications, with SCC being the largest (N = 1,327), BCC mid-tier (N = 767), and pharyngeal carcinoma the smallest (N = 2). The data were extracted from PubMed using search terms specified in online Supplemental Table 1. The database search was performed on May 18, 2024 with no further filters to provide insight into the state and focus of PDT research. G: Summary of clinically approved photosensitizers (PSs) and some widely researched experimental PSs in terms of their chemical- (log P, octanol:water partition coefficient), spectral- (Abs. max., absorption maximum in relation to PDT; ε, molar absorption coefficient at abs. max.), and photochemical properties (Φ_T_, triplet state quantum yield; ϕΔ, singlet oxygen quantum yield) (1) as well as the clinical indications for which the PSs have been approved (2, 3, 4, 5). Abbreviations and trade names: Porfimer sodium (Photofrin); HpD, hematoporphyrin derivative; 5-ALA, 5-aminolevulinic acid (Levulan); PpIX, protoporphyrin IX; mTHPC, m-tetrahydroxyphenylchlorin (Foscan). AlPC(SO_3_)4 is 1 of 3 sulfonated AlPC variants in Fotosens, which constitutes a mixture of AlPC(SO_3_)2–4. The approved indications listed under AlPC(SO_3_)4 pertain to Fotosens. PSs used for superficial conditions are commonly applied topically, while for deeper situated lesions the PS is typically administered via intravenous infusion. H: Optical penetration depth in different human ex vivo tissues plotted as a function of wavelength, showing that longer wavelengths typically penetrate deeper into tissues. The optical penetration depth is defined as the distance at which the light intensity is attenuated to 37% of its initial value. Data were extracted from Stolik *et al.* (6) and replotted. It should be noted that the determinations were performed directly after tissue collection and that the harvested samples were not exsanguinated before spectroscopic measurements. aggr. fibromatosis, aggressive fibromatosis; ax. epid. carcinoma, axillary epidermoid carcinoma; ARMD, age-related macular degeneration; BCC, basal cell carcinoma; NSCLC, non-small cell lung cancer; PWS, port wine stains; SCC, squamous cell carcinoma.

Selective damage by PDT is achieved through target-focused photosensitizing light delivery (commonly through laser-coupled fiber optic cables (9)) that limits exposure to surrounding normal tissues, making PDT more discriminatory than other conventional cancer therapies. An extra layer of selectivity can be conferred by the use of actively targeted PSs (10) as well as passively (1) and actively (11, 12) targeted PS delivery systems. Nanoparticulate PS delivery systems in particular are at the developmental frontier, whereby modern applications are not only directed at the classical cancer and non-malignant superficial ailments but also include neurological disorders and drug-resistant infections (13, 14, 15, 16, 17). As a less invasive intervention, PDT minimizes unwanted long-term side effects, which is also supported by excellent healing properties that preserve organ structure and result in exceptional overall cosmetic outcomes (18). Moreover, the absence of resistance mechanisms in cancer cells presents another important advantage in that it allows the PDT treatment to be repeated many times. These give PDT significant benefits over other clinical modalities.

The advantages are currently offset by the incidence of skin phototoxicity following photoallergic reactions to ambient light after PDT with first- and second-generation PSs (19) and the post-treatment activation of survival signaling in cancer cells (Fig. 2) (20, 21), which can lead to tumor regrowth (25, 26). Skin photosensitization can be tackled by using nanoparticulate PS delivery systems such as liposomes that sterically prevent PS extravasation into the dermis (1). Survival signaling is especially relevant in bulkier tumors with diameters that exceed the optical penetration depth of excitation light (Fig. 2A) (27), which for the clinically accepted PSs (Fig. 1G) ranges between approximately 0.3 and 4.5 mm depending on the type of tissue (Fig. 1H). The ramifications of survival signaling can be curtailed by the co-administration of inhibitors of the survival pathways (22, 23, 27, 28) or ancillary therapies that play in on the PDT-altered biochemical and biological tumor environment, including hypoxic cytotoxins (29), various immunotherapeutic approaches (30, 31, 32), and antitumor lipids (33). Antitumor lipids as pharmacological anticancer adjuvants constitute an emerging and promising niche in the oncological PDT field. As alluded to previously (34), combinatorial cancer therapies are generally preferred over monotherapy to maximize the clinical management of the malignancy. In our view, that also applies to PDT.

**Fig. 2:**
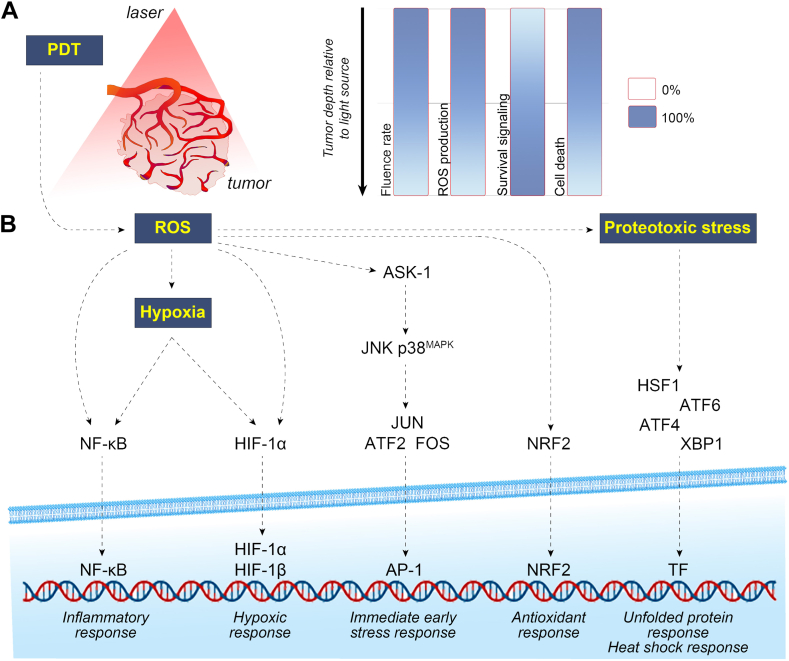
Biological responses in the context of light-photosensitized tissue interactions. A: The 4 most fundamental interrelated phenomena that occur during photodynamic therapy (PDT) in a photosensitized tumor, depicted as a function of tissue depth: the fluence rate (J/cm^2^) decreases with depth in accordance with the Beer-Lambert law, which leads to reduced reactive oxygen species (ROS) generation in the more distal regions of the tumor (relative to the laser probe) given that ROS production is directly proportional to the fluence rate. Tumor cells in the more remote regions consequently suffer less oxidative stress and hence retain the capacity to remediate the PDT-induced damage and activate survival pathways. In contrast, cells situated closest to the incident light experience the most profound ROS production, which debilitates their capacity to execute cell survival mechanisms and in turn leads to cell demise. B: Sublethally afflicted cells activate 1 or more of 6 survival pathways that are triggered by ROS. The pathways are detailed in (20) and their activation has been empirically demonstrated in (21, 22, 23, 24). AP-1, activated protein 1; ASK-1, apoptosis signal-regulating kinase 1; ATF, activating transcription factor; FOS, Fos proto-oncogene; JNK, c-Jun N-terminal kinase; HIF, hypoxia-inducible factor; HSF1, heat shock factor 1; MAPK, mitogen-activated protein kinase; NF-κB, nuclear factor kappa-light-chain-enhancer of activated B cells; NRF2, nuclear factor erythroid 2-related factor 2; TF, transcription factor; XBP1, X-box binding protein 1.

The present work highlights the critical significance of lipids in the response of solid tumors to PDT and elaborates on the opportunities offered by cancer- and PDT-mediated dysregulation of lipid metabolism that can be exploited for improving therapeutic efficacy.

## Deregulation and reprogramming of lipid metabolism in malignant tumors

Metabolic reprogramming and deregulation are recognized hallmarks of cancer (35, 36). Tumor cells reorganize their metabolic pathways as an adaptation to the need for energy to sustain unchecked growth and proliferation, even in a hypoxic and nutrient-poor microenvironment. Rewired bioenergetics pathways that were well-characterized earlier are the elevated rate of aerobic glycolysis (with resulting extracellular lactate making an acidic microenvironment), downregulated oxidative phosphorylation, and upregulated uptake of glutamine (37). More recently, the dysregulated nature of cancer cells in the interconnected network of transcription factors, especially those involved in metabolic and epigenetic reprogramming, has also become recognized (38). Another important, recently elucidated cancer trait is reprogrammed/dysregulated lipid metabolism (39).

The aberration in lipid metabolism is dominated by a shift toward increased de novo lipogenesis, deregulation of exogenous lipid uptake with reduced reliance on dietary and liver-synthetized lipids, increased cellular lipid content, fatty acid oxidation, and lipid-dependent catabolism (40). This secures uninterrupted supply of building segments for membrane formation, signaling molecules, and energy substrates to cancer cells for sustaining their unceasing proliferation and continuous adaptation to a diverging adverse microenvironment. Moreover, stromal and immune cells in the tumor microenvironment also exhibit lipid metabolism reprogramming, which additionally influences tumor functional phenotypes and immune responses (39).

The increased cellular lipid pool in cancer cells is mainly stored within lipid droplets (LDs), which are small organelles whose biogenesis is commonly elevated in cancers, and whose accumulation depends on the activation of the master transcription factor SREBP (sterol regulatory element-binding protein) and mTOR pathways (41). The lipids stored as cholesteryl ester and triacylglycerol within LDs have a key role in reducing cell damage caused by peroxidation of free lipids within the cell (39). Another key function of LDs is protecting cancer cells from ER stress as well as oxidative stress (42).

The transcription factor SREBP is required for transcriptional activation of lipogenic gene expression and is the key upstream regulator of lipid metabolism. In cancer cells SREBP also maintains intracellular cholesterol levels by activating low-density lipoprotein receptor-controlled cholesterol uptake and suppressing its export via ABCA1 transporter (39). Lipid reprogramming has recently been shown to be of critical importance for resistance of tumors to chemotherapy and other types of cancer therapy (37, 40).

## Membrane damage/dysfunction is caused by PDT-induced peroxidation of membrane lipids

### General background

**Fig. 3:**
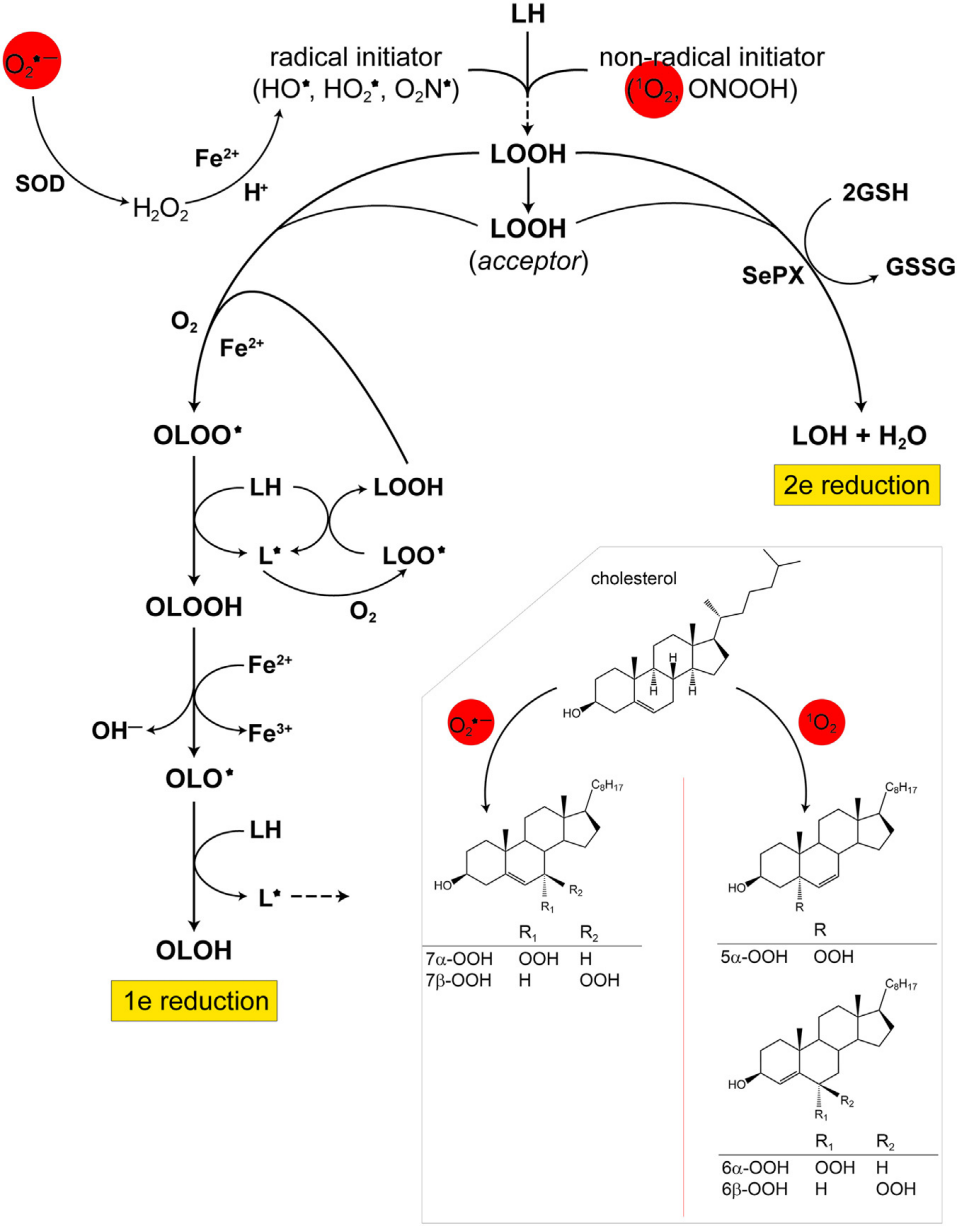
Examples of free radical versus non-radical initiators of membrane lipid peroxidation (LPO). Oxyradical species such as HO^•^ and HO_2_^•^ could derive from type I photodynamic reactions whereas the non-radical ^1^O_2_ is generated by type 2 photodynamic reactions. The species produced specifically by PDT are indicated in red. Photogenerated superoxide anion requires enzymatic conversion to H_2_O_2_ by superoxide dismutase (SOD) and a Fenton reaction to form HO^•^. Radical and non-radical initiators react with membrane lipids (LH) to form lipid hydroperoxides (LOOHs). Various fates of newly formed LOOHs are depicted, including (i) iron-catalyzed 1-electron reduction, which enhances membrane damage; (ii) selenium peroxidase (SePx)-catalyzed 2-electron reduction, which suppresses membrane damage; and (iii) translocation to other (acceptor) membrane compartments, where either type of reductive turnover can occur. Insert: reaction schemes for type I and II photochemical reactions in the context of cholesterol.

Relatively low polarity PSs such as oligomeric hematoporphyrin derivative (Photofrin), benzoporphyrin derivative, metaloided and metallated phthalocyanines, and 5-aminolevulinic acid (ALA)-induced protoporphyrin-IX tend to localize in membrane compartments of cancer cells (e.g., (19, 43)), making these sites highly susceptible to photo-oxidative damage (7, 8, 44). Although damage to membrane proteins can occur, unsaturated lipids such as phospholipids (PLs) (45) and cholesterol (Ch, comprising roughly 30% of membrane lipids) (46) are more prominent targets due to their greater abundance. Lipid photo-oxidation or peroxidation (LPO) can be set off by ROS such as superoxide anion (O_2_^•–^), hydroxyl radical (HO^•^), and singlet molecular oxygen (^1^O_2_) generated by type 1 (electron transfer) and type 2 (energy transfer) photochemical reactions, respectively (47, 48). A general scheme for primary LOOH formation by HO^•^ and other oxyradicals versus ^1^O_2_ and other non-radicals is shown in Fig. 3.

Photogenerated HO^•^, like that arising from light-independent Fenton chemistry (49) involving O_2_^•–^ formation during normophysiological (50) and pathophysiological (51) circumstances, can initiate LPO by abstracting an allylic hydrogen from an unsaturated lipid, e.g., an *sn-2* fatty acyl hydrogen of a PL or a carbon-7 hydrogen of cholesterol (48). Many cancers inherently harbor an elevated labile iron pool (52) and hence exhibit a predilection for Fenton-catalyzed redox reactions. The resulting lipid radical (L^•^) reacts rapidly with O_2_ to give a peroxyl radical (LOO^•^ in general terms or 7-OO^•^ for Ch specifically). The LOO^•^ then induces propagative (chain) LPO by abstracting a hydrogen from a neighboring membrane lipid, thereby becoming a hydroperoxide species (LOOH in general or 7α/7β-OOH for Ch) (48). In contrast to oxyradicals like HO^•^, ^1^O_2_ adds directly to a nearby unsaturated lipid, giving an LOOH with hydrogen retention and allylic shift of the double bond (47, 48, 53). For Ch, the major hydroperoxide species generated by ^1^O_2_ attack is 5α-OOH, but small amounts of 6α/6β-OOH are also formed (48, 54).

Various techniques have been described for analyzing LOOHs, including “bulk-type” methods such as thiobarbituric acid (TBA) and iodometric assays (55, 56). Higher sensitivity, LOOH-differentiating approaches have also been developed, including HPLC with chemiluminescence or electrochemical detection. Reverse-phase HPLC with reductive mode (mercury drop) electrochemical detection [HPLC-EC(Hg)] has gained prominence due to its very high sensitivity, i.e., detection limits of 0.1–0.2 pmol for ChOOHs and 20–30 pmol for PLOOHs (57). Much of the work described below involved the use of HPLC-EC(Hg) to analyze ChOOHs and PLOOHs in lipid extracts from photodynamically stressed tumor cells.

### One-electron reduction of LOOHs with aldehyde end-products

In the presence of suitably chelated ferric iron and a reductant such as ascorbate or superoxide, LOOHs can undergo 1-electron reduction to oxyl radical (LO^•^) intermediates that, either directly or after rearrangement with O_2_ addition to give epoxyallylic peroxyl radicals (OLOO^•^) (58, 59), abstract hydrogens from other lipids, thereby initiating chain LPO (Fig. 3). This is a light-independent post-illumination process that can exacerbate the membrane damaging effects of primary LOOH formation from PDT alone (59). Thus, although primary LOOHs may be detrimental on their own (depending on the levels reached), they could set off secondary (post-illumination) chain LPO that is potentially more damaging and cytotoxic. The after-light process could occur during clinical PDT, but this is still not well recognized by practitioners of this therapy. LPO from 1-electron turnover of primary LOOHs can be suppressed by proximal chain-breaking antioxidants such as α-tocopherol, low level nitric oxide (NO), and butylated hydroxytoluene (BHT) (48). With in vitro photodynamic systems, such agents were found to be effective (though decreasingly so) long after illumination was terminated, indicating that 1-electron reduction had an after-light “momentum” (48). It is now known that the cytotoxicity of such ongoing reactions can far exceed that observed during the illumination period (60). Thus, post-illumination LPO could play a substantial role in the anti-tumor effects of PDT.

Aldehydes such as 4-hydroxy-2-nonenal (HNE) and malondialdehyde (MDA) are known to be important end-products of chain LPO (61, 62). Various studies have shown that HNE and MDA are not inert species but possess significant signaling roles in host cells (61, 62). Depending on levels attained, these aldehydes may exert significant pro-survival versus pro-death effects on mammalian cells undergoing LPO, e.g., malignant cells in tumors exposed to oxidative stress from chemotherapy or radiotherapy (61, 62). Depending on cell type and metabolic status, HNE at relatively low levels can foster cell survival by stimulating antioxidant gene expression via modification of transcription factors such as NRF-2, AP-1, and NF-κB (62), which in and of themselves constitute key survival pathways in sublethally afflicted cancer cells (Fig. 2) (20, 21, 24). However, at higher levels, HNE can begin to induce DNA and protein modifications that lead to cell cycle arrest and apoptotic or necrotic cell death (62). Furthermore, HNE is a specific by-product of omega-6 fatty acid oxidation that, as an “active” aldehyde, will react with any proximal nucleophile (e.g., amines, hydroxyl groups, and sulfhydryl groups) to form covalent adducts, which can compromise biomolecule structure and function (proteins, carbohydrates, nucleic acids) that in turn can manifest as metabolic catastrophe (21). Thus, the eventual outcome can depend on the intensity of chain LPO and the levels of HNE attained in any given cell confronted with oxidative stress. Of related interest, PDT-initiated cell death, promoted by iron-dependent LPO, has been reported to occur via ferroptosis in some systems rather than apoptosis (63).

It should be noted that MDA can serve as a sensitive indicator of LPO extent using the spectrofluorometric TBA assay (55). Moreover, oxidation of omega-3 fatty acids (e.g., docosahexaenoic acid (DHA), C22:6,n-3) results in the formation of other aldehydes such as 4-hydroxyhexanal (64) and carboxyethylpyrrole (65) that exert (patho)physiological and biological functions. Both reactive metabolites play a role in tumor initiation and/or progression (66, 67). In the absence of liquid chromatography (LC) or LC/mass spectrometry (MS) analytic systems these aldehyde adducts serve as surrogates for the detection of LPO in cells and tissues by for instance immunohistochemistry using commercially available antibodies to those adducts (68).

### Two-electron reduction of LOOHs

As an alternative to 1-electron reduction, photogenerated LOOHs may undergo enzyme-catalyzed 2-electron reduction to redox-inert alcohols (LOHs). This is a detoxification process that acts in opposition to toxicity-enhancing 1-electron reduction (Fig. 3). For type 2-generated LOOHs, it is considered a reparative rather than preventative process because unlike HO^•^ from type-1-derived O_2_^•–^/H_2_O_2_, type-2 ^1^O_2_ has no known enzymatic scavengers. Consequently, LOOHs arising from ^1^O_2_ attack need to be removed by reparative enzymes. Glutathione peroxidase-type 4 (GPx4) is the only enzyme known to catalyze the 2-electron detoxification of ChOOHs as well as PLOOHs in biological membranes or lipoproteins (69). Like tetrameric GPx1 (∼84 kDa), which is more abundant in mammalian cells, monomeric GPx4 (∼20 kDa) contains an active site selenocysteine residue (48, 69). These and other SePxs use glutathione (GSH) as a 2-electron donor. While GPx4 can act directly on membrane ChOOHs and PLOOHs, these species, as such, are inert to GPx1. However, after phospholipase action on PLOOHs, released fatty acid hydroperoxides (FAOOHs) can be readily reduced by GPx1/GSH (14). Thus, GPx1 would require this hydrolytic step whereas GPx4 would not require it, suggesting a more direct and rapid cytoprotective effect for the latter. For GPx4-catalyzed reduction of ChOOH species, a broad range of activities has been observed, the first-order rate constants decreasing as follows: 6β-OOH > 7α/β-OOH ≈ 6α-OOH >> 5α-OOH (70). Interestingly, the order of cell-killing potency is the exact opposite of this, with 5α-OOH being the most cytotoxic (70). However, all these ChOOHs are more cytotoxic than PLOOHs because the latter are reductively inactivated by GPx4/GSH much more rapidly than ChOOHs (69). One added note: although 6α- and 6β-OOH are GPx4-inactivated more rapidly than 5α-OOH, their 1-electron turnover, unlike that of 5α-OOH, does not induce chain LPO to any significant extent (71). In this respect, 6α/6β-OOH appears to be unique among the LOOHs described.

### Deleterious effects of LOOH translocation

Damaging LPO due to 1-electron reduction of photogenerated LOOHs is not necessarily limited to their membranes of origin. We now know that ChOOHs, for example, can translocate to other membranes acting as acceptors (Fig. 3). This occurs more rapidly than parent Ch translocation, which is mainly due to the greater hydrophilicity of ChOOHs. First-order rate constants for spontaneous ChOOH transfer were found to decrease in the following order: 7α/β-OOH >> 5α-OOH > 6β-OOH (72), potentially making 7α/β-OOH much more deleterious than 5α-OOH in this context. Sterol carrier protein-2 (SCP-2) is a non-specific intracellular lipid transfer protein that can carry Ch and other lipids as ligands (73). Seminal studies ∼20 years ago revealed that SCP-2 can also transfer ChOOHs between membranes (74). For example, a recombinant SCP-2 accelerated 7α-OOH transfer from liposomes to isolated mitochondria, resulting in chain LPO and loss of membrane potential in the latter (73). Subsequent work showed that SCP-2-overexpressing hepatoma cells took up liposomal 7α-OOH more rapidly than controls, resulting in mitochondrial LPO and apoptosis (75). Intracellular trafficking proteins of the StAR family are specific for Ch and other non-esterified sterol ligands, including ChOOHs such as 7α/β-OOH (76). At least two examples of StAR protein-mediated 7α/β-OOH transfer to mitochondria have been described: one disabling steroid hormone synthesis in testicular cells (77) and the other Ch homeostasis in macrophages (78). Cell death via mitochondria-initiated (intrinsic) apoptosis occurred in both systems after prolonged incubation times. Since the plasma membrane (PM) of mammalian cells is relatively rich in oxidizable Ch and PLs, a PM-localizing PS would generate ChOOHs/PLOOHs in fairly high yields during PDT. SCP-2- or StAR-mediated trafficking of such peroxides to mitochondria could set off membrane LPO that plays a significant role in PDT-induced apoptotic cell death. This is postulated from the evidence described above (73, 74, 75, 76, 77, 78), but it remains to be investigated in an actual PDT setting.

### Nitric oxide as an antagonist of membrane LPO and PDT

**Fig. 4:**
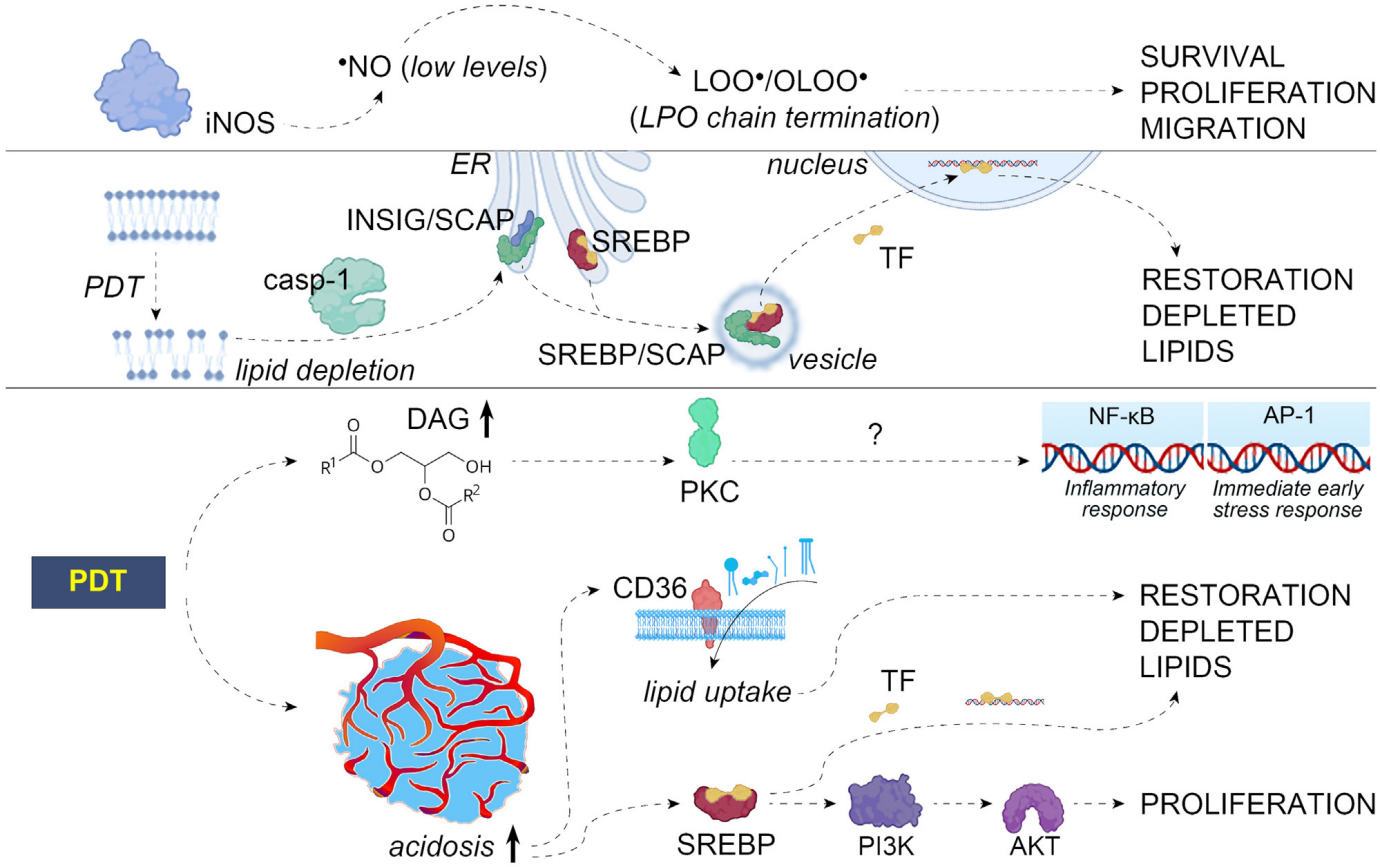
Lipid-related mechanisms that mediate cell survival after photodynamic therapy (PDT). The processes that are described correspond to the following sections in the text. Nitric oxide as an antagonist of membrane LPO and PDT (top panel) addresses how PDT-mediated inducible nitric oxide synthase (iNOS) leads to the production of nitric oxide (^•^NO) that, at low concentrations, facilitates the termination of the lipid (per)oxidation (LPO) chain reaction and cell survival. Activated iNOS and ^•^NO also steer proliferation and cell migration. Hyperoxidative stress repair via the SCAP/SREBP pathway (middle panel) describes how PDT results in the depletion of lipids and repletion of lipids through the SREBP/SCAP pathway, which is initiated by caspase 1 (casp-1) in the endoplasmic reticulum (ER) and completes the transcriptional upregulation of proteins that are responsible for restoring the depleted lipids post-PDT. Abbreviations: INSIG, insulin-induced gene protein; SCAP, SREBP cleavage-activating protein; SREBP, sterol regulatory element-binding protein; TF, transcription factor. Other forms of lipid signaling (bottom panel) highlights the possible additional activation of the NF-κB and AP-1 survival pathways through protein kinase C (PKC), which is activated following a rise in intracellular diacyl glycerol (DAG) content. Also, PDT may exacerbate intratumoral acidity (79) owing to hypoxia and the ad hoc switch to anaerobic respiration in consequence to PDT-induced vascular shutdown (80). The lowering of pH has a bifurcating effect on cell survival. In the one branch, CD36 is upregulated to drive the influx of lipids so as to offset the aforementioned lipid depletion (Hyperoxidative stress repair via the SCAP/SREBP pathway). In the other branch, the increase in acidosis may trigger SREBP signaling and lipid repletion as described in Hyperoxidative stress repair via the SCAP/SREBP pathway whilst also mediating cancer cell proliferation via the PI3K/AKT pathway. AKT, protein kinase B; PI3K, phosphoinositide 3-kinase.

Nitric oxide (NO) is a short-lived bioactive free radical generated by enzymes of the nitric oxide synthase (NOS) family. At a high steady-state level (e.g., ∼1 μM produced by inflammatory macrophages), NO is cytotoxic, whereas at relatively low levels (e.g., < 100 nM), NO can promote survival (Fig. 4), migration, and drug resistance of cancer cells (81). Low-level NO from a chemical donor (SPNO) has been shown to inhibit chain LPO in model membrane systems by intercepting lipid free radical intermediates (82). Subsequent work with breast cancer cells revealed that SPNO-derived NO could suppress PDT-induced LPO in a similar manner, i.e., by intercepting LOO^•^/OLOO^•^ intermediates (Fig. 3), thus acting as a chain-breaking antioxidant (83). More recent studies have demonstrated that cultured breast-, prostate-, and glioblastoma cells sensitized in mitochondria with ALA-induced protoporphyrin-IX can use endogenously generated NO to protect against apoptotic photokilling (84). That inducible nitric oxide synthase (iNOS) was the major source of this NO was established by showing that iNOS knockdown or inactivation with a specific inhibitor enhanced the extent of photokilling (72). These findings were subsequently confirmed at the in vivo level using immunodeficient (SCID) female mice engrafted with human breast MDA-MB-231 tumors (85). A key finding was that iNOS was significantly upregulated after an ALA-PDT challenge and that the resulting NO was mainly responsible for the observed tumor cell hyper-resistance to apoptotic photokilling. Moreover, as observed for MDA-MB-231 cells in vitro (85), and prostate PC3 cells (86), upregulated iNOS/NO signaled for not only apoptotic resistance but also increased proliferative and migratory aggressiveness of surviving cells. Of added importance was the finding that for any cancer cell population, upregulated NO in heavily PDT-targeted cells can diffuse to non- or poorly targeted bystander cells, thereby upregulating iNOS/NO and stimulating aggressiveness in the latter (87). Thus far, these pro-tumor responses to stress-upregulated iNOS/NO appear to be unique to PDT. No similar responses to chemo- or radiotherapy have been reported to date. Although specific inhibitors of iNOS activity might be used as PDT adjuvants to limit the above negative effects, inhibiting iNOS transcription with bromo/extra-terminal domain (BET) inhibitors should be much more effective at lower doses (88). Some of these inhibitors have yielded promising results in anti-cancer clinical trials, albeit not involving PDT (89).

### Summary

Free radical-mediated (chain) lipid peroxidation can play a major cytotoxic role in anti-tumor PDT, particularly for PSs that localize in membrane compartments. However, cancer cells that survive a PDT challenge may upregulate iNOS/NO, which plays a key role in photokilling resistance through NO-mediated inhibition of chain LPO. Upregulated iNOS/NO could also signal for greater proliferative and migratory aggressiveness of surviving cells as well as any proximal non-challenged bystanders. These negative responses to PDT can be counteracted by iNOS activity inhibitors or NO scavengers, but more substantially by BET-type inhibitors of iNOS transcription, which should be more advantageous as clinical PDT adjuvants. We look forward to clinical trials with such inhibitors in the near future.

## PDT-induced lipid repair and signaling responses

### Hyperoxidative stress repair via the SCAP/SREBP pathway

We have shown that the survival of PDT-treated tumor cells depends on the repair mechanism of cellular membranes mediated by the SCAP/SREBP signaling pathway (Fig. 4) responsible for the control of cholesterol and fatty acid metabolism (31). It has been demonstrated that SREBPs are highly upregulated in various cancers (90). The disruption of lipid homeostasis by PDT-inflicted peroxidative lipid injury that produces a drop in cellular levels of cholesterol and other lipids is detected by an ER-situated cholesterol-sensing protein called SREBP cleavage-activating protein (SCAP) (91). This allows SCAP to dissociate from ER-resident insulin-induced gene proteins (INSIGs) facilitating the incorporation of SCAP/SREBP complex into vesicles that transport proteins to the Golgi. At this location, SREBPs are sequentially cleaved by site-1 protease and site-2 protease releasing now-activated transcription factor to travel to the nucleus to activate the transcription of target genes instrumental in restoration of depleted lipids (90, 91). Using the diarylthiazole derivative fatostatin A, which binds to SCAP and blocks the translocation of the SCAP/SREBP complex to the Golgi, we were able to significantly curtail the survival of PDT-treated mouse SCCVII tumor cells (31).

An important role in SREBP activation is played by caspase-1. This caspase, which is activated by inflammasomes assembled upon the induction of membrane damage by PDT (92, 93), is known to trigger the export of SREBPs from the ER (94). Such role is supported by our finding of the additive killing effect in PDT-treated tumor cells incubated with INF-4Es (selective caspase-1 inhibitor) in addition to fatostatin A (31).

### Significance of sphingolipids in cell death and immune signaling

Sphingolipids are a highly bioactive lipid family with a critical impact on PDT signaling (95). Their levels become highly elevated following various stress-inducing treatments, including oncological PDT, due to de novo synthesis prompted by the upregulation of controlling enzymes like dihydroceramide desaturase (96). This results in the increase in tumor concentrations of key sphingolipid molecules, represented by ceramide with its metabolites sphingosine and sphingosine-1-phosphate, which have pivotal roles in cell survival/death and immune surveillance (97). C(18)-ceramide has a central role in cell death signaling leading to mitochondrial apoptosis or other death pathways including necroptosis and mitophagy (98), while blocking its conversion into sphingosine will repress the activity of both lymphoid and myeloid immunoregulatory populations engaged in preventing immune rejection of tumors (95). Ceramide, sphingosine-1-phosphate, and sphingosine were found to become exposed and/or released from the surface of PDT-treated cells exhibiting the capacity of acting as damage-associated molecular patterns (DAMPs), influencing inflammatory processes (inflammasome formation), immune cells, and NF-κB signaling in these cells (95). Various agents altering sphingolipid activity were demonstrated to modify the response of tumors to PDT (96, 99).

### Other forms of lipid signaling

The breakdown of polyunsaturated fatty acids (PUFAs) contained in membrane structures by interaction with PDT-generated LOOHs generates a broad array of short-chain aldehydes and many of them were found to be engaged as signaling molecules in important physiological pathways (62), as mentioned in One-electron reduction of LOOHs with aldehyde end-products.

A variety of other lipid molecules emerging as a result of PDT damage function as autocrine or paracrine signaling molecules, acting either as first messengers (acting extracellularly and serving as ligands to membrane receptors) or as second messengers (intracellular molecules sending signals from membrane to targets). One of such second messengers is diacylglycerol, which typically through protein kinase C activation is engaged in various post-PDT signaling pathways, including NF-κB and AP-1 pathways (Fig. 4) (100). Note that both pathways have been found to be activated following PDT with zinc phthalocyanine (21, 24) and implicated in post-PDT tumor cell survival (Fig. 2) (20). Prostaglandins and lysophosphatidic acid are other important lipid signaling effectors engaged as first messengers in transducing signals integral to PDT responses within tumor cells. Arachidonic acid released from damaged cells is modified to different prostaglandins such as prostaglandin E_2_ (PGE_2_), which binds to membrane receptors to activate downstream signaling pathways as documented with mouse fibrosarcoma cells treated by PDT (101, 102). Lysophosphatidic acid is a widely involved signaling molecule acting as a ligand for G protein-coupled receptors activating multiple pathways that mediate a wide variety of biological actions, including the rise in basal level of intracellular Ca^2+^ as found in PDT-treated human gastric adenocarcinoma cells (103).

Acidosis in the tumor microenvironment consequent to rewired metabolic programming is also responsible for the activation of important signaling routes (39). It results in upregulated levels of CD36 (transporter molecule) promoting exogenous lipid uptake and the formation of LDs. Acidosis also triggers SREBP activation and is responsible for intensified enrollment of the pro-proliferative PI3K/AKT pathway, as demonstrated with PDT-treated tumor cells (104) (Fig. 4).

## Antitumor lipids as adjuvants to PDT efficacy

### Development of antitumor lipids

The existence of striking differences between the lipid metabolism of normal and cancer cells provides attractive therapeutic opportunities for cancer patients that continue to be actively investigated. Strategies under consideration include targeting tumor lipid uptake, blocking lipid synthesis, targeting specifically cholesterol synthesis and uptake, targeting fatty acid oxidation, or aiming at lipid storage in tumor cells (39). An alternative and promising approach is the use of antitumor lipids.

The best-known antitumor lipids are synthetic alkyl phospholipid analogs that have become established as membrane-disruptive anti-neoplastic drugs with demonstrated selective uptake by tumor tissues owing to their accumulation in membrane structures and the endoplasmic reticulum (ER) of cancer cells, and not in the vicinity of their DNA (105). They were shown to interfere with lipid metabolism (particularly de novo phospholipid biosynthesis) and lipid-dependent signaling in tumor cells, affecting lipid composition and cholesterol content. Antitumor lipids exhibit well-defined antiproliferative properties and pro-apoptotic effects selectively in tumor cells (106). Their signaling obstruction impairs critical survival signaling pathways PI3K-Akt and Raf-Erk1/2 (105). These agents are also responsible for a potent and persistent activation of JNK protein kinase that phosphorylates c-Jun transcription factor, which is associated with the activation of the AP-1 transcription factor complex (107). The cytotoxic action of these agents decisively targets the plasma membrane of cancer cells and alters their lipid composition and cholesterol content. This results in the impairment of membrane permeability and fluidity and impedes the function of membrane lipid rafts. The heterogeneous collection of microdomains in the cell membrane, known as lipid rafts, are dynamic assemblies enriched in sphingolipids, cholesterol, saturated phospholipids, and recruited clustered proteins (108). Lipid rafts are also noted for their involvement in the formation of endocytic, exocytic, synaptic, and other vesicles. By disrupting lipid raft composition in the plasma membrane and possibly mitochondrial membrane, antitumor lipids were found to enhance the recruitment and distribution of Fas/CD95 death receptors, which was ensued by the activation of both ligand-dependent and ligand-independent apoptosis of tumor cells (106, 107). It was further revealed that the death receptor recruitment into lipid rafts by alkylphospholipid-based antitumor lipids leads to recruitment of other death signaling molecules into these structures, ending up in the formation of death-inducing signaling complex (DISC) (106). Antitumor lipids are also effective inducers of ER stress, which may promote apoptotic cell death (109). Additionally, antitumor lipids were found to exert a strong anti-inflammatory activity and to possess immunomodulating properties (reducing the expression of MHC class II molecules, inhibiting T cell proliferation, and eliciting a type I interferon response) (110).

**Fig. 5:**
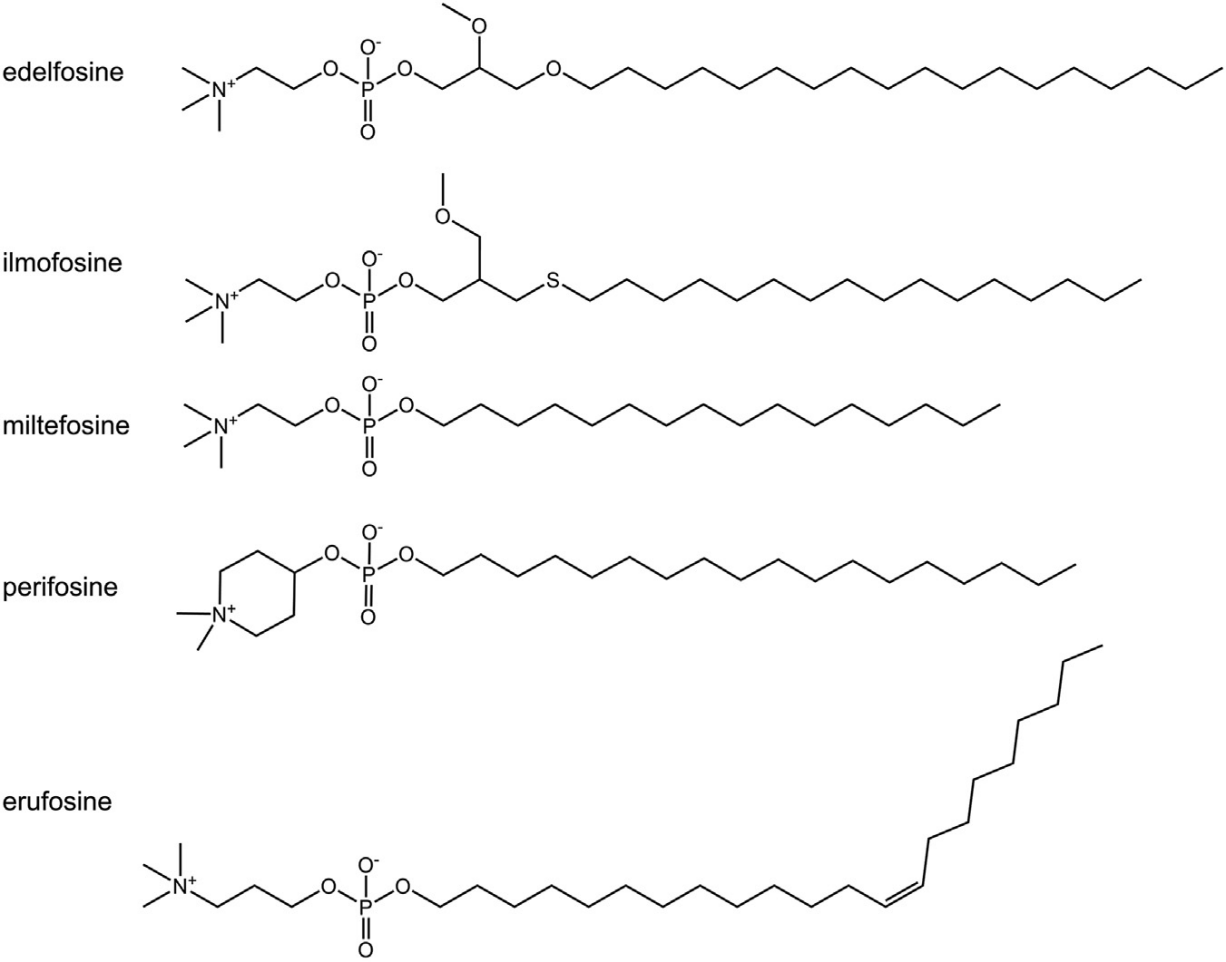
Chemical structure of antitumor lipids that have passed safety and efficacy testing (105).

**Fig. 6:**
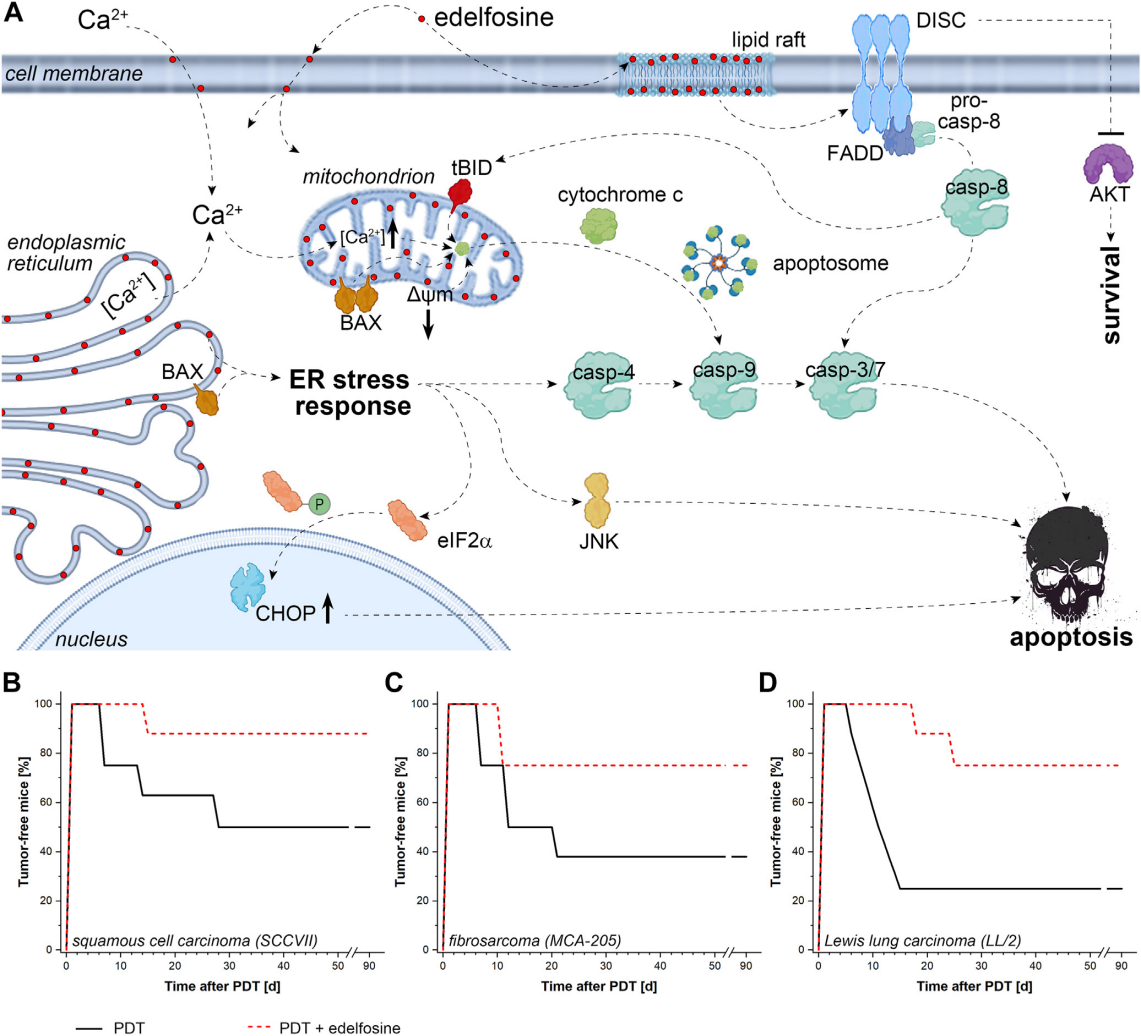
Putative cytotoxicity mechanisms of edelfosine in cancer cells and pharmacodynamic effects on tumor xenografts in vivo. A: Edelfosine () induces apoptosis in cancer cells primarily by integrating into cell and subcellular membranes and disrupting lipid rafts. This leads to altered signaling pathways and impeded survival mechanisms. The compound perturbs membrane integrity that disrupts calcium ion (Ca^2+^) homeostasis, resulting in a rise in cytosolic and subsequent mitochondrial Ca^2+^ concentration [Ca^2+^] (107, 114), which in turn triggers a collapse in mitochondrial membrane potential (Δψm) (115) and subsequent cytochrome c release (116). Additionally, edelfosine translocates to the endoplasmic reticulum (ER) and mitochondria, causing stress that triggers apoptosis through the intrinsic pathway. Key events include membrane defects and activation of Bcl-2-associated X protein (BAX) in the ER and mitochondria, which leads to an ER stress response and cytochrome c release, respectively, and activation of a series of caspases (casp) that culminates in apoptosis. The ER stress response further mediates apoptosis via c-Jun N-terminal kinase (JNK) and C/EBP homologous protein (CHOP) (107, 114, 117). Edelfosine further triggers the Fas ligand (FasL) pathway by altering lipid rafts in the cell membrane, which helps cluster Fas receptors and their associated ligands. This clustering activates the Fas-associated death domain (FADD) proteins, leading to the formation of the death-inducing signaling complex (DISC). DISC then activates caspase-8, initiating the extrinsic apoptotic pathway (107, 117). Caspase-8 triggers truncated BH3-interacting domain death agonist (tBID), which mediates apoptosis through BAX activation and cytochrome c release (118, 119). Finally, edelfosine signaling results in cancer cell survival inhibition through DISC-mediated antagonism of the protein kinase B (AKT) pathway (107, 117). Note that pathways have been truncated to show only key mediators. Some structures were obtained from Biorender.com. B–D: Edelfosine exhibits potent anti-cancer effects in syngeneic xenografts in immunocompetent mice. B: Subcutaneous squamous cell carcinoma (SCCVII) xenografts grown in C3H/HeN mice exposed to peritumorally injected edelfosine (0.1 mg/animal) 24 h after PDT (Photofrin, 6 mg/kg, 630 ± 10 nm, 210 J/cm^2^). C: Subcutaneous fibrosarcoma (MCA-205) xenografts cultivated in C57BL/6 mice exposed to peritumorally injected edelfosine (0.25 mg/animal) 24 h after PDT (Photofrin, 7.5 mg/kg, 630 ± 10 nm, 100 J/cm^2^). D: Subcutaneous Lewis lung carcinoma (LL/2) xenografts cultivated in C57BL/6 mice exposed to peritumorally injected edelfosine (0.25 mg/animal) immediately after PDT (Photofrin, 7.5 mg/kg, 630 ± 10 nm, 150 J/cm^2^). Data in (B–D) were published in (33), furnished by the corresponding author (MK), and used with permission under the Creative Commons Attribution (CC BY) license version 4.0.

The lifetimes of natural alkyl-lysophospholipids in cells are relatively short as they are highly vulnerable to lipase degradation. To become effective therapeutic agents, these compounds have to be rendered metabolically more stable. This was achieved with the synthesis of the first such analog by Günter Kny in 1969 by attaching ether bonds to the C_1_ and C_2_ carbons of the glyceryl backbone (111). The product, 1-octadecyl-2-*O*-methyl-glycero-3-phosphocholine (Fig. 5), was named edelfosine or ET-18-OCH_3_. The absence of ester bonds in this ether lipid renders it inaccessible to degradation by lipases, which are a class of carboxylic esterases that release long-chain fatty acids from natural lipophilic carboxylic esters (112). The uptake of edelfosine in normal cells is significantly lower compared to cancer cells because of the absence of aberrantly programmed lipid metabolism. Consequently, healthy cells are largely spared of the survival-compromising impact of edelfosine, which chiefly affects cancer cells by virtue of a general disruption of cholesterol homeostasis (113). Edelfosine-induced cytotoxicity in cancer cells is detailed at the molecular level in Fig. 6A in addition to the generic outline of molecular signaling provided on alkyl phospholipids in Development of antitumor lipids.

In addition to edelfosine, a number of other alkyl phospholipid analogs were synthetized and tested for their biological activities, and some of these were found promising in the treatment of different diseases in the clinic (120). Edelfosine, ilmofosine, miltefosine, erufosine, and perifosine (Fig. 5) have been deemed safe and promising when tested for their antitumor activity against various malignancies in clinical phase I and phase II trials (105). The first antitumor lipid approved for clinical use was miltefosine for topical treatment of skin metastases in patients with breast cancer (121).

### Oncological PDT combined with antitumor lipid treatment

The antitumor lipids have yielded promising results when used in conjunction with established cancer treatment modalities, including radiotherapy, chemotherapy, and other anticancer therapies (105). This prompted us to investigate the prospects of combining antitumor lipid treatment with PDT. An additional motivation was the fact that both these treatments inflict damage to cellular membranes and instigate ER stress (109, 122). Edelfosine (Fig. 5) was selected as the agent of choice since it is one of the most investigated alkyl phospholipids and considered a prototype molecule of its class (107). The compound is suitable for oral or intravenous administration, with demonstrated low retention in normal tissues. Absence of toxic side-effects were reported with human patients receiving this drug, with no notable cardiotoxicity, no bone marrow or renal injury, and no hepatotoxicity (123).

We first examined cellular stress intensity exhibited by treatment with PDT, edelfosine, or the combined regimen by the expression of heat shock protein 70 (HSP70) on the surface of treated mouse tumor cells. This stress signal, expressed after both PDT only and edelfosine only treatment, was found to be higher after the combined treatment, revealing at least the additive effect on the induction of cellular stress (33). This finding encouraged expectations that the therapeutic impact (in terms of tumor cure rates) with the combined treatment could also be of cumulative nature.

A series of in vivo studies were subsequently conducted using various mouse tumor models treated by Photofrin-based PDT and edelfosine, administered either intratumorally or peritumorally (Fig. 6B–D) (33). Control groups with edelfosine alone showed no significant impact on tumor cure rate. The results with three different types of tumors grown in immunocompetent syngeneic host mice (SCCVII squamous cell carcinoma, MCA-205 fibrosarcoma, and LL/2 Lewis lung carcinoma) demonstrated consistently that edelfosine application resulted in at least doubling of the tumor cure rates obtained with PDT alone. The best results were obtained with peritumoral edelfosine injection, as this route allowed the use of a higher dose (0.25 mg/mouse; 0.48 mmol/mouse) at reduced risk of systemic toxicity compared with intraperitoneal administration.

For the optimal timing, it was demonstrated that edelfosine needs to be delivered after PDT. No beneficial therapeutic effect was obtained with the reversed order (33). There are several possible explanations for this finding. The edelfosine-produced changes in lipid activity and cell membrane damage could be more harmful to targeted tumor cells with already inflicted injury from PDT. Another possibility is that edelfosine is most effective when potentiating already existing PDT-inflicted membrane lesions and obstructing the repair mechanisms. Additional possible explanations include the interactions of edelfosine-triggered cell death signaling with progressing PDT-instigated survival signaling (20). A further scenario could feature impaired restoration of lipid homeostasis by edelfosine-mediated downregulation of the lipid removal from cells interfering with PDT-induced SCAP/SREBP membrane repair pathway activity. On the level of stress signaling, edelfosine-induced ER stress may override survival signaling in the ER prompted by PDT and rewire signal transduction activity from prosurvival/cytoprotective to death-promoting. Further investigations into the mechanistic aspect of the interaction between PDT effects and antitumor lipid action would be of obvious interest.

The above-described findings with edelfosine can be considered as compelling albeit preliminary proof-of-principle for the use of anti-cancer lipids as adjuvants to tumor PDT. Importantly, the therapeutic impact of oncological PDT appears especially vulnerable to manipulations in the tumor lipid microenvironment. The prospects for exploiting potential impacts warrant further investigation.

## Conclusions

The importance of photo-induced changes in membrane and other cancer cell lipid structures by PDT in relation to therapy outcome has been largely overlooked and underestimated. When interpreting events underlying PDT-mediated therapeutic gain, taking into account the dramatically altered nature of lipid composition and metabolism in cancer cells compared to normal tissues is generally omitted. As emphasized in Membrane damage/dysfunction is caused by PDT-induced peroxidation of membrane lipids, the burden of inflicted molecular damage after PDT is far greater with lipids than with other intracellular biomolecules. This pertains not only to structural impairment through e.g., deleterious redox propagation but even more importantly to the functioning of cellular signal transduction pathways because of critical roles played by lipid signaling molecules.

The abovementioned factors render the response of tumors to PDT critically vulnerable to maneuvering with the tumor lipid microenvironment. This realization inspires designing novel approaches for potentiating the therapeutic impact of PDT by exploiting the potentials offered by lipid manipulation. One such approach, as elaborated in the present article, is the adjuvant use of antitumor lipids.

At doses not affecting normal tissues, adjuvant antitumor lipids have the potential to markedly increase PDT-mediated tumor cures. This can be attributed to their selective uptake by tumor tissues and their tumor-selective anti-proliferative and pro-apoptotic properties. These agents exert well-evidenced interference with lipid metabolism and lipid-dependent signaling in cancer cells with impact on membrane lipid composition and cholesterol content (88).

Additional approaches targeting lipid functions that could be considered for use in conjunction with PDT include blocking tumor uptake or synthesis of lipids or specifically cholesterol, inhibiting fatty acid oxidation, targeting lipid storage in tumor cells, or selective delivery of catalytic co-factors for lipid redox reactions (e.g., expansion of labile iron pool).

## Data availability

All data can be provided by the corresponding author.

## Supplemental data

This article contains supplemental data.

## Conflict of interest

The authors declare that they have no conflicts of interest with the contents of this article.
